# Antenatal Corticosteroid Exposure in Late Preterm Infants: Clinical Considerations in Diabetic Mothers

**DOI:** 10.7759/cureus.110222

**Published:** 2026-06-04

**Authors:** Hannah Daniel, Esther S Kim, Tristan Grogan, Juanita Lewis, Kalpashri Kesavan, Supriya Bavisetty

**Affiliations:** 1 Department of Pediatrics, University of California, Los Angeles Mattel Children's Hospital, Los Angeles, USA; 2 Department of Neonatology, Olive View-University of California, Los Angeles Medical Center, Sylmar, USA; 3 Department of Medicine Statistics Core, University of California, Los Angeles David Geffen School of Medicine, Los Angeles, USA; 4 Department of Neonatology, University of California, Los Angeles David Geffen School of Medicine, Los Angeles, USA; 5 Department of Pediatrics, Olive View-University of California, Los Angeles Medical Center, Sylmar, USA

**Keywords:** antenatal corticosteroids, betamethasone, late preterm infants, maternal diabetes, neonatal hypoglycemia, neonatal respiratory support, preterm birth, respiratory outcomes

## Abstract

Objective

The objective of this study was to evaluate the association between antenatal corticosteroid (ACS) administration and respiratory and metabolic outcomes among late preterm infants, with a secondary exploratory analysis of infants of diabetic mothers.

Methods

We performed a retrospective cohort study of 85 neonates born between 33 0/7 and 35 6/7 weeks’ gestation at a county hospital to investigate neonatal outcomes according to ACS exposure. Outcomes included respiratory support requirement, duration to room air, hypoglycemia, and hospital length of stay.

Results

Among 85 infants, 18 (21%) received no ACS, 24 (28%) received one dose, and 43 (51%) received two doses. ACS exposure was not associated with increased hypoglycemia (p = 0.163) or hospital length of stay (p = 0.647). Among infants requiring respiratory support (n = 30), greater ACS exposure was associated with fewer days to room air (Spearman’s rho = -0.37, p = 0.044).

Conclusions

In this small retrospective cohort, ACS exposure was associated with shorter time to room air among late preterm infants requiring respiratory support, without increased risk of hypoglycemia. These findings are exploratory and hypothesis generating, particularly for infants of diabetic mothers.

## Introduction

The use of antenatal betamethasone for women at risk of late preterm delivery (34 to 36 weeks’ gestation) is an evolving area of perinatal care aimed at improving neonatal outcomes [1,2]. Traditionally, antenatal glucocorticoids have been administered to pregnant women at risk for early preterm delivery (before 34 weeks) due to strong evidence showing that these steroids significantly reduce adverse neonatal outcomes, such as respiratory distress syndrome and intraventricular hemorrhage [1,3-5]. However, the administration of these steroids after 34 weeks has been less common, primarily due to a perceived lack of data and the belief that late preterm infants generally thrive without additional interventions [1,6-8]. Despite near-survival rates comparable to term infants, late preterm infants face a higher risk of neonatal and childhood health complications, raising the question of whether antenatal corticosteroids (ACS) could benefit this population [9-11].

The 2016 Antenatal Late Preterm Steroids (ALPS) trial was a multicenter, randomized study evaluating the benefits of betamethasone for women at 34 0/7 to 36 5/7 weeks’ gestation at high risk of late preterm delivery [1]. Infants exposed to betamethasone were significantly less likely to require respiratory support within the first 72 hours of life compared with those receiving a placebo [1]. Additional benefits included lower rates of transient tachypnea of the newborn, decreased surfactant use, reduced bronchopulmonary dysplasia, and less need for immediate postnatal resuscitation [1]. These findings aligned with the earlier Antenatal Steroids for Term Elective Caesarean Section (ASTECS) trial, which demonstrated reduced neonatal intensive care unit (NICU) admissions for respiratory complications among term infants receiving ACS before elective cesarean delivery [12]. Although the ALPS trial noted increased neonatal hypoglycemia, rates were comparable to typical late preterm populations and were not linked to adverse outcomes, supporting continued glucose monitoring for all late preterm infants [5]. Following this evidence, the American College of Obstetricians and Gynecologists issued guidance recommending a single course of corticosteroids for women between 24 0/7 and 33 6/7 weeks at risk of preterm birth within seven days [5]. Standard regimens include two 12 mg intramuscular doses of betamethasone 24 hours apart or four 6 mg doses of dexamethasone every 12 hours [5]. ACS are administered to accelerate fetal organ maturation and are associated with reduced neonatal morbidity and mortality [5].

The administration of steroids to diabetic mothers with gestational age greater than 34 weeks remains a critical question [2]. Infants born to diabetic mothers are already at increased risk of hypoglycemia in the neonatal period due to hyperinsulinemia from increased glucose exposure in utero, as well as at increased risk of respiratory complications, with studies suggesting that maternal hyperglycemia and fetal hyperinsulinemia inhibit lung maturation and surfactant production [13-15]. Limited studies have investigated ACS administration in this subpopulation of women at risk of late preterm delivery, with consideration for use between 34 0/7 and 36 6/7 weeks [5]; the ALPS trial excluded women with pregestational diabetes, and only about 10% of its study population had gestational diabetes [1]. Given the lack of data regarding ACS for individuals with pregestational diabetes and the observed increased risk of neonatal hypoglycemia, the Society for Maternal-Fetal Medicine currently recommends against the use of late preterm corticosteroids in pregnant patients with pregestational diabetes mellitus [2,16].

Overall, ACS therapy plays a vital role in improving neonatal outcomes by enhancing fetal maturation, reducing neonatal complications, and supporting the health and survival of preterm infants [5]. There remains an inconsistency in the literature regarding the risks and benefits of ACS for late preterm neonates, particularly among those born to infants of diabetic mothers [17]. In a small county hospital setting with a Level III NICU, we performed a retrospective analysis to elucidate the impact of adequate corticosteroid treatment on respiratory outcomes in late preterm infants compared with those partially or without steroid treatment. Our primary objective was to evaluate the association between ACS exposure and respiratory outcomes and true hypoglycemia risk in late preterm infants. Our secondary, exploratory objective was to examine outcomes among infants of diabetic mothers, given that concerns about hypoglycemia have historically precluded this population from receiving ACS.

## Materials and methods

This single-center retrospective cohort study was performed in a safety-net hospital in California with a Level III NICU that serves a diverse, medically underserved population and delivers roughly 800-1,000 infants each year. We included 85 neonates, all eligible neonates born between 33 0/7 and 35 6/7 weeks’ gestation from January 2023 to October 2024. Infants at 33 weeks were included because they are managed clinically within the same late preterm workflow at our institution and thus follow identical respiratory and glucose monitoring protocols. Infants with major congenital anomalies were excluded.

The project received institutional review board exempt status. Data were manually extracted from the electronic medical record and included ACS doses received, gestational age, birth weight, maternal diabetes status, delivery mode, APGAR scores, respiratory support requirements, and hypoglycemia. Manual chart extraction was performed using a standardized data collection protocol to ensure data quality. Spot checks of randomly selected records were conducted by senior study personnel, and data elements were cross-referenced against multiple available data sources to verify accuracy and completeness. Formal inter-rater reliability testing was not performed and is acknowledged as a limitation. Glucose values were obtained via point-of-care testing per hospital protocol. Hypoglycemia was defined as blood glucose <45 mg/dL in the first 24 hours of life [14]. Respiratory support data included the level of support required and the duration of respiratory support. Infants of women with both pregestational and gestational diabetes were included.

Continuous variables were summarized using means and standard deviations and, when helpful, medians and interquartile ranges. Categorical variables were summarized using frequencies and percentages. Outcomes and baseline characteristics were compared across ACS dose groups (the no ACS group received no ACS, the partial ACS group received only one dose, and the complete ACS group received two doses) using one-way ANOVA for continuous variables and chi-square or Fisher’s exact tests for categorical variables, as appropriate. The timing of ACS relative to delivery could not be analyzed due to incomplete documentation. Respiratory support was evaluated both as any respiratory support versus none and, among infants requiring support, as lower-level support (nasal cannula/continuous positive airway pressure (CPAP)) versus higher-level support (noninvasive positive pressure ventilation (NIPPV)/synchronized intermittent mandatory ventilation (SIMV)). Escalation criteria at our institution include persistent tachypnea, increased work of breathing, recurrent desaturations, or inadequate ventilation despite CPAP, prompting a transition to NIPPV/SIMV. Among infants who required respiratory support, the association between increasing ACS dose and day of life weaned to room air was evaluated using Spearman’s rank correlation. Analyses were performed using R version 4.4.3 (R Foundation for Statistical Computing, Vienna, Austria), and two-sided p-values < 0.05 were considered statistically significant. Given the modest sample size and baseline differences in birth weight, delivery mode, and maternal diabetes status, multivariable adjustment was not feasible and is acknowledged as a limitation.

## Results

Among the 85 infants in our study, 18 (21%) received no steroid doses, 24 (28%) received a partial one-dose steroid course, and 43 (51%) received a full two-dose steroid course. There was no significant difference in gestational age (p = 0.88), small for gestational age status (p = 0.404), gender (p = 0.353), or ethnicity (p = 0.788) among these three groups. Gestational age did not differ significantly across ACS dose groups. When analyzed as a continuous variable, gestational age showed a nonsignificant trend toward variation (p = 0.088), but this association disappeared when gestational age was categorized into week-based groups (p = 0.223), indicating no meaningful differences in gestational age distribution by ACS exposure. There was significant variation in the number of ACS doses received based on infant birth weight, where lower birth weight infants were significantly more likely to have received ACS (p < 0.001), reflecting clinical practice patterns in our institution. Infants born by cesarean section were significantly more likely to have received ACS than those born vaginally (p = 0.017) (Table 1).

**Table 1:** Distribution of clinical and demographic characteristics by ACS dose group Values are shown for the full sample and for each ACS dose category. Statistical tests evaluate differences across the three groups, with corresponding test statistics and p-values reported. ACS, antenatal corticosteroids

Characteristic	Overall (n = 85)	Zero ACS doses (n = 18)	One ACS dose (n = 24)	Two ACS doses (n = 43)	Test statistic	p-Value
Gestational age, weeks					χ²(6) = 7.6	0.223
33 weeks	13 (15.3%)	0	4 (16.7%)	9 (20.9%)		
34 weeks	26 (30.6%)	6 (33.3%)	5 (20.8%)	15 (34.9%)		
35 weeks	32 (37.6%)	8 (44.4%)	12 (50.0%)	12 (27.9%)		
36 weeks	14 (16.5%)	4 (22.2%)	3 (12.5%)	7 (16.3%)		
Gestational age, continuous	34.6 (0.8)	34.9 (0.7)	34.5 (0.8)	34.4 (0.9)	F(2, 82) = 2.5	0.088
Small for gestational age (Yes)	10 (11.8%)	2 (11.1%)	1 (4.2%)	7 (16.3%)	χ²(2) = 2.2	0.404
Infant of diabetic mother (Yes)	29 (34.1%)	12 (66.7%)	5 (20.8%)	12 (27.9%)	χ²(2) = 11.1	0.004
Male	48 (56.5%)	8 (44.4%)	16 (66.7%)	24 (55.8%)	χ²(2) = 2.1	0.353
Hispanic	66 (77.6%)	15 (83.3%)	19 (79.2%)	32 (74.4%)	χ²(2) = 0.63	0.788
Birth weight (g)	2295.8 (443.3)	2633.2 (470.3)	2365.0 (320.8)	2115.9 (403.0)	F(2, 82) = 11.3	<0.001
Delivery mode (CS)	49 (57.6%)	12 (66.7%)	8 (33.3%)	29 (67.4%)	χ²(2) = 8.1	0.017

Twenty-nine infants (34%) were born to diabetic mothers with either pregestational or gestational diabetes. Diabetic mothers were significantly less likely to have received ACS (p = 0.004). Forty-eight (56%) of infants experienced hypoglycemia in the first 24 hours of life. There was no significant difference in the probability of neonatal hypoglycemia based on the number of ACS doses received (p = 0.163). Among hypoglycemic infants, nadir glucose values did not differ by ACS exposure (p = 0.807). There was no significant difference in hospital length of stay based on ACS administration (p = 0.647) (Table 2).

**Table 2:** Association of maternal diabetes status and ACS dose with hypoglycemia in the first 24 hours of life Hypoglycemia was defined as blood glucose <45 mg/dL in the first 24 hours. Neither maternal diabetes status nor ACS dose group was significantly associated with hypoglycemia. Maternal diabetes status: χ²(1) = 0.403, p = 0.525. ACS dose group: χ²(2) = 3.623, p = 0.163. All associations were assessed using Pearson chi-square tests. ACS, antenatal corticosteroids

Characteristic	No hypoglycemia	Hypoglycemia	p-Value
Maternal diabetes: No	23/56 (41.1%)	33/56 (58.9%)	0.525
Maternal diabetes: Yes	14/29 (48.3%)	15/29 (51.7%)	
ACS dose: 0	11/18 (61.1%)	7/18 (38.9%)	0.163
ACS dose: 1	11/24 (45.8%)	13/24 (54.2%)	
ACS dose: 2	15/43 (34.9%)	28/43 (65.1%)	

Fifty-five infants (65%) remained on room air following delivery, while 30 infants (35%) required some form of respiratory support. ACS exposure was not associated with the incidence of respiratory support (p = 0.93). Among infants requiring support, mean days to room air did not differ significantly across ACS groups by ANOVA (p = 0.101). However, increasing the ACS dose was significantly associated with fewer days to room air by Spearman correlation (ρ = -0.37, p = 0.044) (Table 3).

**Table 3:** Days to room air among infants requiring respiratory support by number of ACS doses Among the 30 infants who required respiratory support, the mean days to room air did not differ significantly across ACS dose groups by one-way ANOVA, F(2, 27) = 2.502, p = 0.101. However, increasing the ACS dose was associated with shorter time to room air based on Spearman rank correlation, ρ = -0.37, p = 0.044. For correlation analyses, Spearman’s rho (ρ) is reported as the test statistic. ACS, antenatal corticosteroids

ACS doses	n	Mean days to room air (SD)	Median (IQR)
0	7	6.1 (5.9)	4.0 (2.5, 7.5)
1	8	5.8 (2.4)	5.0 (5.0, 6.5)
2	15	2.9 (2.9)	2.0 (1.0, 4.5)

## Discussion

Our finding of an association between greater ACS exposure and shorter duration of respiratory support aligns with existing literature suggesting improved respiratory outcomes for late preterm infants who received ACS, including the original ALPS trial [1]. While the ANOVA comparing mean days to room air across groups was not significant, the Spearman correlation was significant, indicating that higher ACS exposure was associated with fewer days of respiratory support, though this reflects correlation rather than causation. One cross-sectional study found that in the nine months following the dissemination of the ALPS trial, ACS administration among infants born in the late preterm period in the United States more than doubled, with an associated 9% decrease in assisted ventilation among these infants, suggesting true benefit outside of the context of a clinical trial [11,18]. Another retrospective study of 2,103 late preterm infants born in Turkey found that ACS administration was associated with decreased risk of respiratory distress syndrome, transient tachypnea of the newborn, length of stay in the NICU, and need for mechanical ventilation [8]. Importantly, in our cohort, ACS exposure was not associated with the incidence of respiratory support, only with the duration among infants who required it. This distinction is consistent with the ALPS trial and subsequent observational studies.

Our findings regarding the lack of a significant increase in hypoglycemia risk are corroborated by a 2021 secondary analysis of infants studied in the original ALPS trial [19]. The secondary analysis, including 2,609 infants, found that hypoglycemia generally resolved in the first 24 hours of life for all infants, regardless of corticosteroid exposure, and time to resolution was shorter among infants in the betamethasone group [19]. These findings support the notion that although hypoglycemia was more common among infants exposed to ACS, it did not have a significant impact on the affected infants [19]. In our cohort, ACS exposure was not associated with increased hypoglycemia using the AAP threshold of <45 mg/dL [14].

Some studies have sought to understand risks and benefits specifically for infants of mothers with diabetes, given limited data on this population from the original ALPS trial. Contrary to our findings, a 2024 retrospective cohort study of 24,323 late preterm infants born to mothers with pregestational diabetes found that ACS administration was associated with worse outcomes, including increased assisted ventilation, NICU admission, and surfactant use [13]. It has been suggested that ACS may not have the expected benefits for infants of diabetic mothers because maternal hyperglycemia with resultant neonatal hyperinsulinemia may inhibit glucocorticoid-mediated lung maturation, a mechanism observed in animal models [20].

One retrospective cohort study of 123 infants of diabetic mothers from 2021 found that late preterm ACS exposure was associated with an increased rate of hypoglycemia; however, hypoglycemia was defined as <60 mg/dL in the first 24 hours [21], a threshold higher than the AAP recommendation [14]. Their study also found no association between ACS exposure and increased hospital length of stay, NICU admission, need for IV dextrose, or glucose <40 mg/dL, again suggesting that any increased hypoglycemia risk may not be clinically significant [21]. Another retrospective cohort study of 453 diabetic women with late preterm deliveries found that ACS administration was not associated with increased neonatal hypoglycemia risk; however, a complete course of ACS was associated with decreased supplemental oxygen use, mechanical ventilation, and respiratory distress syndrome, further corroborating our findings [20]. Prior studies have reported mixed findings in diabetic pregnancies, and our study contributes real-world data but cannot resolve these conflicting results.

This study has several limitations. The modest sample size with three-arm distribution and single-site design limits generalizability, and the nonrandomized nature of ACS exposure introduces the potential for confounding by indication. Infants who received ACS differed from those who did not in factors such as birth weight, delivery mode, and maternal diabetes status, and the sample was insufficient to support adjusted multivariable analyses. The sample size was insufficient to support adjusted multivariable analyses, introducing baseline imbalances that could influence observed associations. Our subgroup of diabetic mothers represented a secondary objective, and, despite being underpowered, we felt it was important to analyze due to the significantly reduced likelihood of receiving ACS. The incomplete documentation of ACS timing prevented assessment of dose timing effects. The inclusion of infants at 33 weeks may further limit applicability to strictly late preterm populations. Finally, the study does not capture long-term neurodevelopmental outcomes, an important consideration given emerging data on potential later-life risks [7,18]. Existing evidence regarding ACS use in diabetic pregnancies remains inconsistent, underscoring the need for larger, rigorously designed studies to clarify risks and benefits in this population. At the same time, the real-world safety-net context of this cohort represents a meaningful strength, as it reflects clinical practice in medically underserved settings where data are limited.

Despite these limitations, the study provides hypothesis-generating evidence that ACS exposure may be associated with shorter respiratory support duration without increasing hypoglycemia risk in a diverse, safety-net population. Larger, prospective studies, particularly those focused on diabetic pregnancies, are needed to clarify risks and benefits.

## Conclusions

ACS exposure was associated with shorter time to room air among infants who required respiratory support and was not significantly associated with hypoglycemia in this small retrospective cohort. These findings suggest that ACS may have a respiratory benefit without a clear increase in short-term hypoglycemia risk; however, the results should be interpreted cautiously because ACS exposure was not randomized and differed by maternal diabetes status, birth weight, and delivery mode. Given the limited sample size, particularly among infants of diabetic mothers, these findings should be considered hypothesis generating rather than practice changing.
